# The Porifera Ontology (PORO): enhancing sponge systematics with an anatomy ontology

**DOI:** 10.1186/2041-1480-5-39

**Published:** 2014-09-08

**Authors:** Robert W Thacker, Maria Cristina Díaz, Adeline Kerner, Régine Vignes-Lebbe, Erik Segerdell, Melissa A Haendel, Christopher J Mungall

**Affiliations:** Department of Biology, University of Alabama at Birmingham, Birmingham, USA; Museo Margarita, Boca de Rio, 6304 Venezuela; CR2P, UMR 7207 CNRS-MNHN-UPMC, Département Histoire de la Terre, Muséum National d’Histoire Naturelle, Bâtiment de Géologie, CP48, 57 rue Cuvier, 75005 Paris, France; Department of Medical Informatics and Clinical Epidemiology, Oregon Health & Science University, Portland, USA; Genomics Division, Lawrence Berkeley National Laboratory, Berkeley, CA USA

**Keywords:** Morphology, Taxonomic identification, Phylogenetics, Evolution

## Abstract

**Background:**

Porifera (sponges) are ancient basal metazoans that lack organs. They provide insight into key evolutionary transitions, such as the emergence of multicellularity and the nervous system. In addition, their ability to synthesize unusual compounds offers potential biotechnical applications. However, much of the knowledge of these organisms has not previously been codified in a machine-readable way using modern web standards.

**Results:**

The Porifera Ontology is intended as a standardized coding system for sponge anatomical features currently used in systematics. The ontology is available from http://purl.obolibrary.org/obo/poro.owl, or from the project homepage http://porifera-ontology.googlecode.com/. The version referred to in this manuscript is permanently available from http://purl.obolibrary.org/obo/poro/releases/2014-03-06/.

**Conclusions:**

By standardizing character representations, we hope to facilitate more rapid description and identification of sponge taxa, to allow integration with other evolutionary database systems, and to perform character mapping across the major clades of sponges to better understand the evolution of morphological features. Future applications of the ontology will focus on creating (1) ontology-based species descriptions; (2) taxonomic keys that use the nested terms of the ontology to more quickly facilitate species identifications; and (3) methods to map anatomical characters onto molecular phylogenies of sponges. In addition to modern taxa, the ontology is being extended to include features of fossil taxa.

## Background

Porifera (sponges) are sessile, aquatic, multicellular animals that lack true organs and a nervous system. Instead, sponges contain loosely aggregated cells that can differentiate into a variety of cell types and produce diverse skeletal structures. These skeletal elements can be comprised of proteinaceous spongin, chitin, collagen, calcium carbonate and/or silica, depending on the species. Traditional sponge systematics defines sponge taxa by recognizing particular sets of morphological features described in sources such as *Systema Porifera* [1]. Although these features have been well characterized in the *Thesaurus of Sponge Morphology* [2, 3], and used in pioneering Artificial Intelligence (AI) classification systems [4–6], the terms that are used to describe sponge morphology have not previously been organized into the framework of a modern ontology.

Sponges are conspicuous components of most benthic marine ecosystems such as shallow coral reefs, mangroves, mesophotic reefs, and deep water environments [7]. Sponges play critical roles in these ecosystems, contributing to global cycling of carbon and nitrogen, stabilizing (but also eroding) coral reef frameworks, and hosting incredibly diverse communities of macroscopic and microscopic symbionts [8, 9]. Furthermore, sponges have therapeutic potential and other human applications due to their ability (or that of their symbionts) to synthesize various unusual compounds [10] and therefore present a wealth of biotechnological application opportunities.

**Figure 1:**
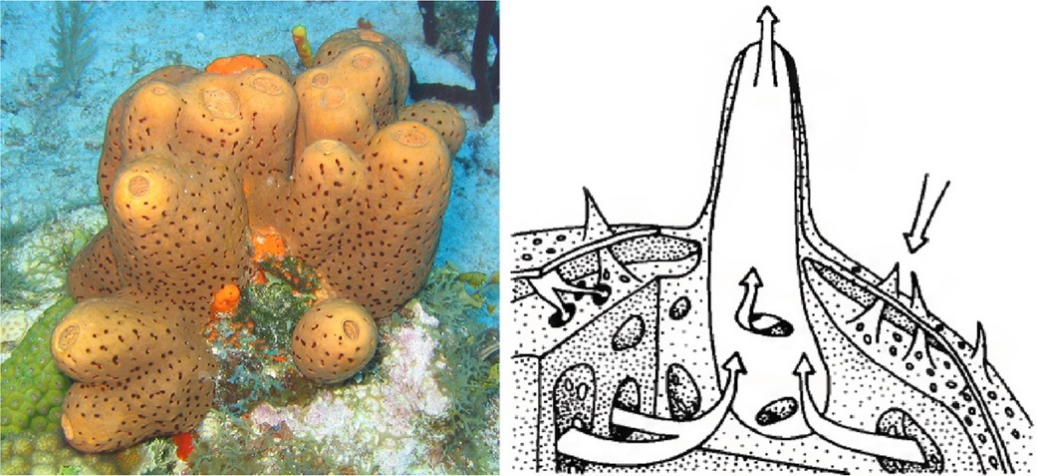
**Marine sponges like** ***Agelas conifera*** **(a, left) contain an aquiferous system that pumps water through the sponge body (b, right; from** [2] **).**

Sponge life depends on the flow of water through an aquiferous system (Figure 1), with water flowing into the body through incurrent openings (ostia), through a network of canals that are lined by internal epithelium-like cells (pinacocytes), into chambers lined by collared, flagellated cells (choanocytes), and out of the body through excurrent openings (oscules). Choanocytes closely resemble choanoflagellates, a group of unicellular eukaryotes that are among the closest relatives to multicellular animals [11]. Sponges are of interest to evolutionary biologists studying the origins of multicellularity in animals and the origins of the nervous system [12]. Despite having no neurons or synapses, some sponges have a nearly complete set of post-synaptic protein homologs [13]. Likewise, sponges possess the elements of the cadherin and β-catenin complex that are critical for cellular adhesion in bilaterian tissues [11]. Therefore, a more formal representation of poriferan anatomy would enable more complex queries across a diversity of taxa in search of protein, network, and biological processes that have regulated the evolution of multicellularity and the nervous system.

### Ontologies for evolution

Biological and biomedical ontologies are structured vocabularies that provide consistent names and textual definitions for anatomical structures, biochemical entities, processes and functions associated with gene products, and many other kinds of biological features. With the success of the Gene Ontology (GO) [14], ontologies have become common in biology [15–17] and more recently the systematics and evolutionary phenotype communities have begun to use them for character description [18–20]. Through the use of description logic formalisms underpinning the Web Ontology Language (OWL) [21] (a World Wide Web Consortium standard) they facilitate semantic reasoning within and across domains that can be performed by computers. Many freely available, open-source ontologies have been developed that provide terms suitable for annotating, describing, and integrating a wide array of biological data [22].

Ontologies are not the same as databases or taxonomic keys – however, ontologies can be used as an enhancement to database systems and keys, both as a standard terminology, allowing different database systems to interoperate, and as a logical extension, allowing domain knowledge to be encoded in a way that enhances query capabilities or data integrity. For example, the GO provides a stable identifier denoting the biological process of apoptosis – different databases can use this same identifier for describing genes involved in apoptosis, allowing integration of data from multiple databases covering genes in a variety of species. Furthermore, the knowledge that ‘apoptosis’ is a kind of ‘cell death’ is encoded in the ontology, which means queries for genes involved in ‘cell death’ will return genes described as being involved in apoptosis.

One important type of biological ontology is the anatomy ontology. Anatomy ontologies typically include relationships between structures, such as the relationship of parthood between a hand and a limb. In sum, these relationships form a graph structure that can be used to enhance database queries or bioinformatic analyses. For example, a database query for genes expressed in the limbs can return genes expressed in different parts of the limb (such as the hands) or deeper in the part-hierarchy (e.g., in the distal part of the finger). Anatomical ontologies are also used to standardize character-state descriptions in evolutionary databases, such as, for example with the Phenoscape knowledge base [18].

The fundamental unit of these anatomical graph structures are *classes* (also known as *terms*). Each class represents a distinct anatomical feature and is typically assigned a unique identifier that provides a key with which it may be cross-referenced to other ontologies or databases. The open nature of commonly used bio-ontologies allows terminology and definitions to be re-used from other ontologies with which they overlap, reducing duplication of effort and promoting orthogonality.

Some anatomy ontologies cover a specific taxon – for example, the Drosophila Anatomy Ontology (DAO) [23] Others are applicable to a wide range of taxa – for example, the Plant Ontology (PO) [24] or the Uberon anatomy ontology [25], which covers metazoans. Until now, the major focus of anatomy ontologies has been plants and bilaterians, with no representation of the unique biology of sponges – whilst Uberon includes structures applicable across animals, the focus of the ontology is chordates, with the intention of federating with other metazoan ontologies.

For a period in the 1990s, sponges were amongst the domains modeled in pioneering Expert System research [4, 6]. In particular, SPONGIA was a rule-based system for classifying the species of a sponge given as input a set of character descriptors and measurements [26]. Expert systems have some similarities with ontologies – both are concerned with knowledge representation and classification of concepts and data. In fact, expert systems research has largely fragmented into different data science domains, including Bayesian networks (for representing and reasoning with probabilistic knowledge) and description-logic based ontologies (for representing and reasoning with boolean knowledge). One consequence of these advances in information science is that first-generation expert systems do not interoperate with modern information systems. Ontologies provide a means of encoding domain knowledge in an application-independent way.

The present study initiates an ontological approach to the morphology of Porifera by interpreting and organizing the major anatomical characters developed by sponge taxonomists as summarized by the *Thesaurus of Sponge Morphology* [2].

## Results and discussion

### Ontology contents

We constructed the Porifera ontology (PORO) as a Web Ontology Language (OWL) ontology using the *Thesaurus of Sponge Morphology* as a primary source. The ontology primarily focuses on anatomical structures, but includes other kinds of entities of interest to Poriferan biologists – for example, traits and chemical entities. Each anatomical entity is represented using an OWL class which is uniquely identified by a URI (uniform resource identifier) in the OBO Library “PORO” identifier space. In this paper, we provide examples of classes using short forms of these URIs - for example, PORO_0000017 identifies the class ‘spicule’. The full URI of this class is “http://purl.obolibrary.org/obo/PORO_0000017”, which resolves to an OWL document rendered as a human-readable web page using the OntoBee system. In the current release, the ontology contains 625 classes unique to PORO (i.e., not imported from other ontologies), with 27 classes imported from other ontologies. Of the 625 unique classes, 519 have definitions that have been sourced from the *Thesaurus of Sponge Morphology* [2].

### Upper level classification

The ontology follows the Common Anatomy Reference Ontology (CARO) [27] upper level, making use of standard upper-level terms such as “organism substance” and “material anatomical entity” to structure the ontology. Due to the fundamental biology of sponges, many CARO classes such as “organ” were not used. In contrast to other anatomy ontologies, many (50%) of the anatomical classes in the ontology are subtypes of ‘acellular anatomical entity’ (for example, spicules and fibers). At this time, only a minimal subset of CARO is being used (9 classes). CARO is currently being refactored and extended, and the development of PORO will serve as a use case for this work. For example, CARO may include a generic class for representing anatomical chambers, which may serve as the parent class for choanocyte chamber in PORO.

### Body plan

A sponge body consists of three distinct functional layers around an aquiferous system that can consist of a combination of pores, incurrent and excurrent canals, choanocyte chambers, and exhalent atria (Figure 1). The most interior layer (the choanoderm) contains choanocytes, which are the collared, flagellated cells that form the choanocyte chambers (PORO_0000025). The most exterior layer (the pinacoderm) contains the epithelial-like pinacocytes (PORO_0000023), which are tightly connected to each other and line the internal canals and external surfaces. Sandwiched between these two layers is the mesohyl (PORO_0000002), an extracellular matrix composed primarily of galectin, collagen, fibronectin-like molecules, dermatopontin, and other polypeptides; the mesohyl contains cells (microbial and eukaryotic) and skeletal elements (collagen, spongin, chitin, and/or minerals) [2, 28]. The choanoderm, pinacoderm, and mesohyl are represented as separate non-overlapping partitions of the sponge body through the use of OWL General Class Inclusion axioms (GCIs). The sponge aquiferous system can be very simple, as in the small, sac-shaped, asconoid (PORO_0000149) bodies of some Calcarea, or extremely complex, as in the leuconoid (PORO_0000028) structures found in most other sponges [29].

### Acellular structures comprising the architecture of sponges

Characteristic features of many sponges are spicules (PORO_0000017), which form the skeleton of the organism in most cases. Spicules can be composed of calcium carbonate, silica, or spongin. They may also be classified by size (megascleres or microscleres). The primary means of classifying them is by their morphological, and in particular, symmetric structure. For example, a triaxone (PORO_0000602) is a spicule with 3 axes and 6 rays. This can be modeled precisely in OWL using a construct called a *cardinality constraint*. In the Manchester Syntax variant of OWL, this is written as:

‘triaxone’ EquivalentTo

spicule and

(has_component exactly 3 ‘ray axis’) and

(has_component exactly 6 ‘ray’)

**Figure 2:**
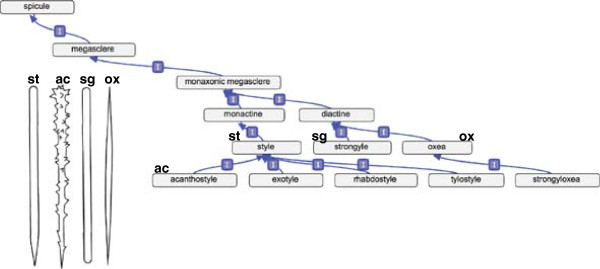
**Ontology visualization showing a portion of the spicule hierarchy (with many terms omitted for space reasons).** The graph visualization is drawn using the OBO-Edit Graph View plug-in for Protégé 4. Inset are examples of spicules including, from left to right, a style (st), an acanthostyle (ac), a strongyle (sg), and an oxea (ox).

Figure 2 shows a subset of the spicule hierarchy, focused on ‘acanthostyle’.

Fibers are another architecturally important class, with fiber skeletal arrangements usually being dendritic or reticulate. Spicules can be embedded within the fibers or echinate (protrude from) the exterior of the fibers. The overall pattern of spicule and fiber distribution within a sponge is termed the skeletal arrangement, with many defined categories (for example, a dendritic or a reticulate arrangement, but there are several other possible patterns). These different skeleton types are represented in the ontology.

### Cell types

**Figure 3:**
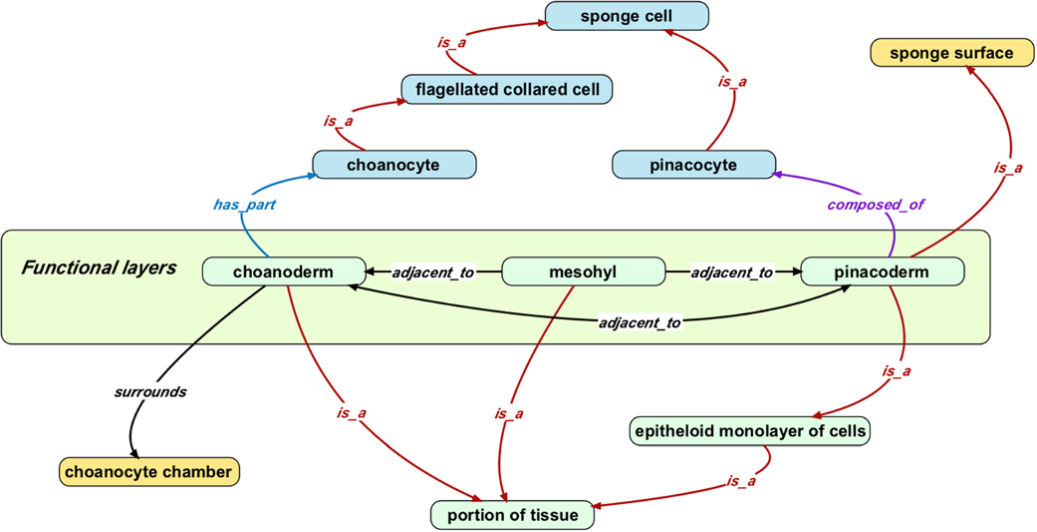
**A subset of PORO illustrating the three layers of structures comprising a whole organism and some of the cell types that comprise these layers.** The mesohyl is a gelatinous layer sandwiched between the external pinacoderm and internal choanocyte-lined surface (choanoderm). We re-use the CARO class ‘portion of tissue’ for these layers, although some debate exists about whether these features constitute true tissues.

We decided to keep cell types within PORO, rather than add them to the central OBO cell type ontology (CL) [30], as these are relatively few in number and are largely specific to sponges. Examples include ‘bacteriocyte’ (PORO_00001062), ‘actinocyte’ (PORO_0000107) and ‘choanocyte’ (PORO_0000003). The latter is of particular interest to evolutionary biologists due to their proposed homology to choanoflagellates. Many sponges lack true epithelia with basement membranes, so we introduce a class ‘epithelioid cell’ (PORO_0000004) rather than re-using the Cell Ontology class for ‘epithelial cell’. However, many of the gene products required for epithelia are found in Porifera [31], and Homoscleromorpha have basement membranes and true epithelia [29], so in these cases use of the CL class may be justified. Figure 3 shows some of the cell types in PORO, together with the tissue layers in which they are located.

### Chemical entities and proteins

We reuse classes from the Chemical Entities ontology CHEBI [32] for chemical structures of relevance – for example, ‘calcium carbonate’ (CHEBI_3311), ‘biogenic silica’ (CHEBI_64389) and ‘aragonite’ (CHEBI_52239). In some cases, these are connected from other parts of the ontology via a ‘composed from’ relation. For example, ‘calcareous spicule’ and ‘calcium carbonate exoskeleton’ are composed of ‘calcium carbonate’. Many sponge taxonomists use biochemical markers and lipid profiles as descriptors, so we anticipate extending this part of the ontology in the near future.

### Qualities and traits

We include qualities and traits used by sponge taxonomists in the ontology. For example, under ‘relationship to substrate’ we have ‘sessile’ (PORO_0000526), ‘rooted’ (PORO_000050) and ‘endolithic’ (PORO_0000284). In future versions of the ontology some of these terms will be contributed back to the PATO phenotype and trait ontology [33].

### Applications and future directions

Studies of poriferan systematics have increased considerably in recent times, with the number of researchers studying sponges doubling in the past two decades [29]. The application of molecular approaches to sponge taxonomy has revolutionized and considerably improved our understanding of the diversity and complex evolutionary history of this group [34, 35]. However, the pace at which molecular systematics generates information about the phylogenetic diversity of sponges has not been matched by a corresponding acceleration of our understanding of the morphological and functional dimensions of this biological diversity. The incorporation of an ontological approach to organize and connect structural, functional, genetic, and gene expression concepts will allow us to improve this situation.

The phylum Porifera contains over 8,000 accepted species [1], but at least 6,000 additional species are thought to exist based on surveys of museum collections [36]. When integrating morphological and molecular datasets, most studies of sponge systematics find high support for morphological classifications at the species level, indicating the ability of morphological characters to distinguish between sponge species [29, 34, 37–39]. However, there is often a lack of resolution at the genus and family levels, suggesting that morphological characters are often homoplasic [29, 37]. This low phylogenetic resolution within orders is not surprising since there are relatively few morphological characters available for analyses and since these characters can be phenotypically plastic. Recently, Morrow et al. [40, 41] demonstrated that some homoplasic characters may actually represent distinct morphological traits that are described by a single term (e.g., “acanthostyle”, Figure 2).

A major question in the development of multi-species anatomy ontologies is whether ontological terms should be designed with an assumption of the homology of anatomical structures [20]. In constructing PORO, we took an explicitly pragmatic approach and made no assumptions that these ontological terms refer to evolutionarily homologous characters. Although there are many known instances of homoplasy throughout sponge systematics [37, 40, 41], our current goal is to reflect anatomical terms as they have been used in recent and historical literature. For example, the term ‘actine’ refers to the ray of a spicule [2]. For sponges bearing calcareous spicules, actines do not contain an axial filament, while for sponges bearing siliceous spicules, actines do contain an axial filament [42, 43]. Although it is clear that ‘actine’ is referring to a feature of two evolutionarily distinct types of spicules, the concept of ‘actine’, that of a ray [2], provides a practical term when describing this feature. In future studies, we plan to use the PORO ontological framework to describe homoplasic characters and hope to provide a higher degree of resolution when describing particular anatomical features. Greater precision in naming morphological features might allow sponge biologists to create less ambiguity in character states, yielding less homoplasy. While the question of how much homology to build into an ontology is debated [44], it is common for multi-species ontologies to include structures that are not explicitly determined to be homologous, and we have previously used this strategy for other anatomical ontologies [25].

A practical concern when identifying sponges is poor specimen preservation. In some cases, critically important morphological features can be difficult to determine, yielding difficulty in assigning taxonomic names using existing, primarily bifurcating, identification keys. By integrating the morphological ontology with taxonomy, we hope to enable the creation of polytomous identification keys that can function with incomplete data sets. By using the Porifera ontology to annotate images of sponge morphology, we will facilitate the proper identification of anatomical features.

In the future, we plan to extend the core ontology to include extinct taxa. For example, Archaeocyatha are fossil sponges from the Cambrian era that lack spicules. Their functional biology is only deduced from a theoretical model [45], but they appear to share many characters with modern Porifera. Kerner et al. [46] standardized descriptions of the morphological characters of Archaeocyatha, building a descriptive knowledge base of illustrated and clearly defined terms. We are currently adding these characters into the Porifera Ontology and explicitly connecting anatomical terms between fossil and modern taxa. By linking the skeletal elements of Archaeocyatha to those of sponges, we hope to enhance our understanding of the functional biology of Archaeocyatha as an analog of sponges.

It is important to note that PORO is not a complete database, knowledge base, or application in its own right – however, PORO can form the terminological and deductive knowledge backbone of such a system. PORO is complementary to classification aids such as SPONGIA [26], which is a powerful expert system able to infer the species of a sample based on the answers to questions concerning descriptive characters. Indeed, in constructing that system, the authors noted:

“The simple work of character definition in the domain model turned out to be a non-trivial task. A thesaurus of terms for sponges that was to contain an important consensus among the European experts in sponge systematics was in the pipeline and its preliminary versions were available to us. However, the current vocabulary of “our” expert was not always standardized according to the previous consensus [26]”.

PORO could be used directly as the domain model used in systems such as SPONGIA and its successors. One intriguing possibility is the encoding of the taxonomic classification rules of SPONGIA directly in OWL, allowing the use of modern Description Logic reasoners. Given that OWL is expressive enough to encode classification rules involving conjunction and disjunction of either symbolic or quantitative characters, it may seem that this would be easily achieved. However, one challenge is that the MILORD II framework used by SPONGIA makes use of many-valued logic, reasoning with uncertainty and non-monotonic reasoning, all of which are outside the scope of OWL. One research possibility would be to combine Bayesian and ontological reasoning, as has been done in disease classification [47].

Finally, a major effort is underway by the *Next-Generation Phenomics* research team [48] to automate the process of deriving character matrices from published species descriptions. As part of our future work, we will use the ontology to annotate text and to standardize the names of morphological characters across various research groups over the past 200 years of sponge taxonomy. In addition, the *Phenomics* team is seeking to automate character recognition using image processing software. It will be crucial to annotate reference images with terms from the ontology to calibrate this novel imaging system.

## Conclusions

There are a number of ontologies covering taxa such as plants, fungi and bilaterians, but the Porifera ontology is the first ontology dedicated to a non-bilaterian metazoan. Because many terms used in sponge taxonomy and systematics have Porifera-specific meanings, we created the structure of the Porifera ontology from existing resources, primarily the *Thesaurus of Sponge Morphology* [2]. We will revise and expand PORO to accommodate new concepts and relationships as we use the ontology to build character matrices for modern and fossil taxa. By accelerating our ability to describe and understand sponge morphology, we seek to reconcile differences between morphological and molecular approaches to poriferan systematics. We hope that this integrative approach to taxonomy and systematics will inspire investigators working with invertebrate and microbial taxa to add value to their morphological datasets by placing the characters used to describe additional taxonomic groups into an ontological framework.

## Methods

### Bottom-up ontology development

When building an anatomy ontology, it is possible to take a ‘top-down’ approach or a ‘bottom-up’ approach (or some combination thereof). With a top-down approach, the creator starts with upper-level categories and gradually introduces more specific classes. With a bottom-up approach, the creator starts with the terms of interest (which are typically more specific) and builds them into a hierarchy, gradually ‘working-up’ to the root.

With PORO we took a bottom-up approach. We started with the online version of the *Thesaurus of Sponge Morphology* [2] and used a Perl script to generate a skeleton ontology in OBO format. This was adjusted using a text editor and OBO-Edit [49], and then translated into OWL and edited using the Protégé 4 ontology editor [50] (http://protege.stanford.edu). The translation retained the textual definitions obtained from the thesaurus, as well as annotations on the definitions referencing the source material. These are represented in the OWL ontology as axiom annotations.

For Protégé editing, we make use of a number of plugins, including one for annotating images (https://github.com/balhoff/image-depictions-view), OBO-Edit style productivity assistance tools (https://github.com/balhoff/obo-actions) and the OBO-Edit Graph View plugin for Protégé (http://code.google.com/p/obographview/).

The editors of the ontology met through meetings organized by the Phenotype Research Coordination Network (RCN), where the ontology was reviewed and biologists were provided with training in ontology building and reasoning.

We make use of the HermiT reasoner as part of the ontology development process [51]. Due to the use of OWL features such as cardinality constraints, we cannot use the faster reasoners that operate over restricted profiles of OWL, but due to the current relative small size of the ontology, reasoning can be performed dynamically.

As well as using reasoning within Protégé, we run reasoner checks as part of an automated build process, using the OBO Ontology Release Tool (http://code.google.com/p/owltools/wiki/OortIntro) executed within a Continuous Integration server [52]. This server also checks for common problems that can occur during Protégé editing, such as duplicate labels, equivalent classes, or classes having multiple text definitions.

### Availability

PORO is always available in OWL from the OBO Library permanent URL http://purl.obolibrary.org/obo/poro.owl. The content of the ontology is available under a CC-BY license (http://creativecommons.org/licenses/by/2.0). Further details can be obtained from the project website https://code.google.com/p/porifera-ontology/.
